# Quantitative Structure Activity Relationship study of the Anti-Hepatitis Peptides employing Random Forests and Extra-trees regressors

**DOI:** 10.6026/97320630013060

**Published:** 2017-03-31

**Authors:** Gunjan Mishra, Deepak Sehgal, Jayaraman K Valadi

**Affiliations:** 1 Shiv Nadar University, Gautam Budha Nagar, Uttar Pradesh 201314, India;; 2 Center for modelling and simulation, Savitri Bai Phule Pune university, Pune, Maharastra 411007, India;

**Keywords:** Anti-Hepatitis peptide (AHP), Quantitative structure activity relationship (QSAR), Descriptors, Extra Tree and Random Forests algorithm

## Abstract

Antimicrobial peptides are host defense peptides being viewed as replacement to broad-spectrum antibiotics due to varied advantages. Hepatitis is the commonest infectious disease of liver, affecting 500 million globally with reported adverse side effects in treatment therapy. Antimicrobial peptides active against hepatitis are called as anti-hepatitis peptides (AHP). In current work, we present Extratrees and Random Forests based Quantitative Structure Activity Relationship (QSAR) regression modeling using extracted sequence based descriptors for prediction of the anti-hepatitis activity. The Extra-trees regression model yielded a very high performance in terms coefficient of determination (R2) as 0.95 for test set and 0.7 for the independent dataset. We hypothesize that the developed model can further be used to identify potentially active anti-hepatitis peptides with a high level of reliability.

## Background

According to WHO, infectious disease of liver, hepatitis, caused by Hepatitis A-E virus, affects 500 million globally and has gravely affected the developing nations of South –Asia [1]. Current anti-virals are inept to face the challenge of rapidly changing virus and co-infections thus emphasizing need for novel diagnostic and therapeutic alternatives, antimicrobial peptides being one of them. Antimicrobial peptides, peptides of usually 11-100 amino acids, are synthetic or endogenously secreted in body as an immune response against bacteria, protozoa, fungi and viruses. The success of these ‘host defense peptides’ has been translated till clinical trials [2]. The mode of action is varied as it blocks 1) attachment of virus 2) viral replication machinery 3) Virus release to neighboring cells and also alter host immune responses. This multiple mode of action allows wide spectrum activity and is also successful in cases of drug resistance. Thus, they are being viewed as an alternative to broad-spectrum antibiotics. However, the wet lab validation and tailoring of the peptide activity is both resource and time consuming. Hence, in-silico modeling methodologies such as QSAR prove to be a boon for the analysis of bio-chemo-physical property and activity of peptides [3].

Quantitative structure activity relationship (QSAR) is the study of relationship between biological activity of the compounds as a function of their physio-biochemical properties expressed in terms of descriptors [4]. It has found wide ranging applications in fields of computational biology and drug discovery, as it offers a cheaper and faster alternative to conventional methods of experimentally determining the activity of compounds. Recent methodologies from machine learning field like Random Forests and Extra-trees algorithm have been found to perform very well in QSAR modeling. The studies including the QSAR of the antimicrobial peptides are also reported in literature [5].

Antimicrobial peptides active against hepatitis are called as antihepatitis peptides (AHP). In our previous work, we reported the collection of AHPs and performed the sequence based modelling of AHP [6]. In present paper, sequence based descriptors will be used to establish quantitative structure-activity relationships using machine learning based algorithms. We hypothesize that the QSAR based studies on the peptides will bring an insight on the therapeutic potential of the peptides and accelerate the drug discovery process.

## Methodology

We collected data from publically available resources and obtained 153 peptides sequences along with their IC50 activity. We then extracted 8809 descriptors including 1) amino acid composition 2) dipeptide composition 3) tripeptide composition 4) conjoint triad descriptors 4) Quasi sequence order descriptors 5) sequence order descriptors 6) Composition, Transition, Distribution descriptors 7) Pseudo amino acid composition (PAAC) and 8) Amphiphilic pseudo amino acid composition (APAAC) using ProtR [7]. We then employed the Information Gain filter method, as implemented in WEKA [8]. For this purpose, we first thresholded the activities and converted the original regression problem to classification problem. The information gain was hence calculated and we found 157 descriptors to be informative. Subsequently, the dataset was randomly split into 80% training set, 10%test set and 10% independent set.

The algorithms used were:

### 1) Random Forests based regression

Random Forests in machine learning are a popular tool for the classification and regression tasks. It is a collection of randomly generated independent decision trees. The first type of randomness introduced is in form of bootstrap sampling in each tree. The second type of randomness is in terms of subset selected for the best split in each node (n<N). The random forest built with these two randomness has been found to be very robust and accurate [9]. The use of Random Forests for QSAR has been well reported in the literature [10].

### 2) The Extra- Trees algorithm

This algorithm is very similar to random forests algorithm where large numbers of the decision tree models are built. However, there are two key differences. The first difference lies in complete random splitting of the descriptors at node. The second difference is that every tree is grown with the entire dataset instead of bootstrap sampling [11].

## Result & discussion

In present work, we have employed the algorithm of Extra-Trees and Random Forests for the QSAR of Anti-hepatitis peptides (AHPs) and found the Extra-Trees model to show promising results.

For this purpose, 153 peptides were collected through a comprehensive survey. The total of 8809 descriptors were extracted using the ProtR [7]. To find the best contributing informative descriptors, we have used Information Gain filter method. 157 descriptors were found to be informative and were taken for further analysis. Subsequently, the dataset was randomly split into 80% training, 10% test set and 10% independent set.

We have used R based codes for the implementation of Random Forests and Extra-Trees algorithm. The parameter viz. number of trees employed in the forest and the subset of the feature for each tree and each split, were optimally fine-tuned so as to get best performance measure in terms of coefficient of determinism (R2). The models were also used for the independent dataset to assess the predictive performance of the developed models. The results are provided in the Table 1.

Though we have used similar parameters for fine-tuning of the two algorithms, the vast difference of result can be understood in light of variance-bias trade-off. The methodology of the Extra- Trees involves the complete random splitting at the node, thus introducing extra variance. Moreover, the use of the entire dataset instead of bootstrap sampling minimizes the bias. The higher grade of randomness during the training yields more independent trees and thus further decreases the variance. Extra- Trees work by decreasing variance and increasing bias simultaneously. This makes the Extra-Trees methodology superior to its counterpart algorithms [11].

We hence, report that the method demonstrated its effectiveness in finding the IC50 values of the anti-hepatitis peptides with best coefficient of determinism reported as 0.95 for test set and 0.7 for the independent dataset with Extra-Trees algorithm. Thus, we hypothesize that the model obtained can be used for building the QSAR towards anti-hepatitis activity.

## Conclusion

The drug resistance and adverse side effects of current antihepatitis treatments has implied to look for newer therapies, antimicrobial peptides (AMP) being one of them. Antimicrobial peptides are new replacement for broad spectrum antibiotics and a few studies have been reported for QSAR analysis of antimicrobial peptides [5],[12], [13]. However, no study has been reported for the QSAR of anti-microbial peptide active against hepatitis. We hypothesize that the study pertaining to antihepatitis spectrum activity will help identify factors which are responsible for selective action against different classes of microbes and can be implied for various diagnostic and therapeutic applications.

Various algorithms for QSAR modelling e.g. multiple linear regression, partial least squares, Support Vector Machines and Random Forests hybridized with evolutionary and heuristic techniques like ant colony optimization, particle swarm optimization, artificial bee colony algorithm, genetic algorithm etc. are reported to be used in the literature to build efficient QSAR models. However, there is a need to enhance the accuracy, interpretability and confidence in the computational models. As a future prospective, the success of QSAR model can help in development of the potent and selective inhibitors for the treatment of strain specific hepatitis. This will also aid in accelerating the disease –oriented drug discovery pathways.

## Figures and Tables

**Table 1:** Results of the regression on the AHP data

Regressors	R2(Test set)	R2 (Independent set)
Extra-Trees	0.95	0.72
Random Forests	0.774	0.548
